# Aortoesophageal fistula complicated by mycotic aneurysm secondary to endoscopic procedures: A case report

**DOI:** 10.1002/ccr3.7690

**Published:** 2023-07-17

**Authors:** Shokrollah Hafezeftekhari, Farzaneh Khoroushi, Hossein Bozorgi

**Affiliations:** ^1^ Department of Radiology, Faculty of Medicine Mashhad University of Medical Sciences Mashhad Iran

**Keywords:** AEF, aortoesophageal fistula, mycotic aneurysm, upper GI bleeding

## Abstract

Aortoesophageal fistula (AEF) is a rare cause of upper gastrointestinal bleeding that often receives little attention in the emergency department. The classic presentation includes the Chiari triad of central chest pain, sentinel arterial bleeding, and subsequent evacuation after an asymptomatic period. For patients suspected of having AEF, a CT scan with IV contrast is the preferred diagnostic modality. In our patient, the presence of an aortic wall outpouching, ectopic gas, periaortic fat stranding, and leukocytosis, even in the absence of fever and positive blood culture, suggested mycotic aneurysm with AEF. The unique aspect of this case report is the occurrence of AEF as a rare complication of endoscopic procedures, which should be considered. Treatment options for AEF include surgery and thoracic endovascular aortic repair (TEVAR). TEVAR is a good option for stabilizing the patient's condition and reducing mortality in the short term. Once the patient's condition is suitable for AEF repair surgery, surgical intervention can be performed.

## Graphical abstract

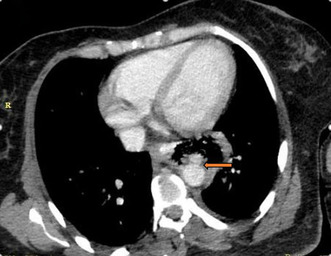

## 1 INTRODUCTION

Gastrointestinal bleeding is one of the leading causes of hospitalization. The most common causes of upper gastrointestinal bleeding are gastric ulcer, gastritis, esophageal varices, and Mallory–Weiss syndrome. However, there are also less common causes that often receive little attention in the emergency department, such as aortoesophageal fistula (AEF).^1^ AEF is a rare but life‐threatening cause of upper gastrointestinal bleeding, with a mortality rate of 77% with appropriate treatment and 100% without treatment.^2^

AEF can be primary or secondary. The primary type is caused by various factors, including thoracic aortic aneurysm, foreign body, esophageal cancers, and radiotherapy. The secondary type is usually caused by complications of surgeries performed on the aorta or esophagus, and can also occur as a result of the implantation of surgical grafts.^3^

Clinical symptoms of AEF can range from asymptomatic patients to very critical patients. Classic patients present with the Chiari triad of central chest pain, sentinel arterial bleeding, and fatal bleeding after an asymptomatic period.

Various diagnostic methods have been introduced for the diagnosis of AEF, which can be selected based on the patient's clinical condition and vital signs. A dynamic CT scan may be a faster option for stable patients after sentinel bleeding, but patients with clinically unstable conditions should undergo immediate surgery.^1^

Various treatment methods have been proposed for AEF, including surgery and interventional radiology. Thoracic endovascular aortic repair (TEVAR) is a good option for stabilizing the patient's condition and reducing mortality in the short term until the patient undergoes reconstructive surgery.^4^

In the present study, the patient experienced upper gastrointestinal bleeding due to the development of an AEF fistula following endoscopic procedures.

## 2 CASE PRESENTAITION

A 36‐year‐old Asian woman with no underlying medical conditions, but with a history of abdominoplasty surgery, presented to the hospital with persistent nausea and vomiting for 2 weeks. After supportive treatment and upper GI endoscopy, a significant narrowing was observed in the distal esophagus caused by an esophageal ring, preventing the scope from passing through. No evidence of an obvious ulcer was found during the endoscopic evaluation of the esophagus. Malignancy was ruled out after a biopsy of the esophageal stricture, and the stricture was ultimately dilated by balloon through the scope. The patient was discharged in good general condition with regular medication.

Ten months later, the patient returned to the hospital with persistent nausea and vomiting and received supportive treatment. During endoscopy, which was performed due to the patient's history of an esophageal ring, esophagitis was observed in the esophageal mucosa, and the patient underwent re‐dilation by bougie.

**FIGURE 1:**
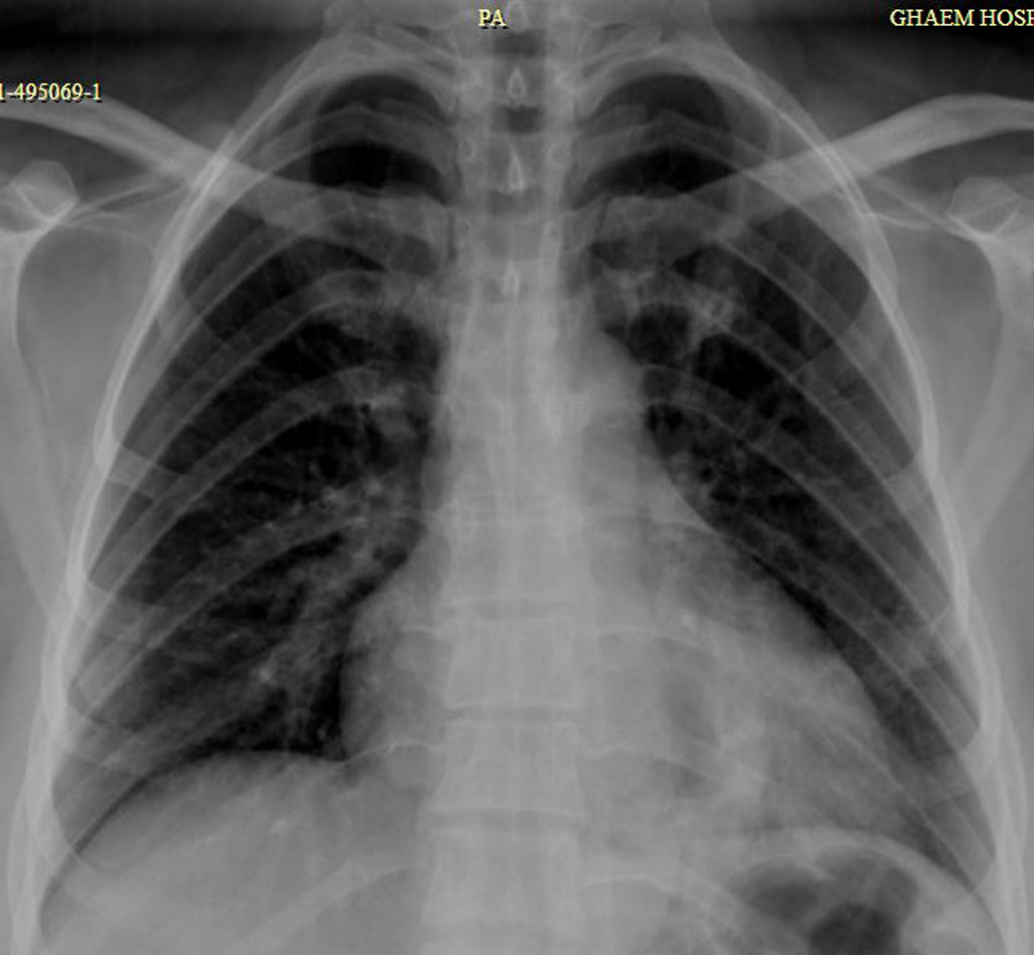
PA chest radiograph shows which does not reveal any mediastinal widening or other pathological findings.

Following bougienage, the patient experienced chest pain accompanied by fever and leukocytosis, leading to suspicion of esophageal perforation and mediastinitis. To further investigate, the patient underwent a chest x‐ray and CT scan with IV and oral contrast. The chest x‐ray did not show any pathological findings (Figure 1).

**FIGURE 2:**
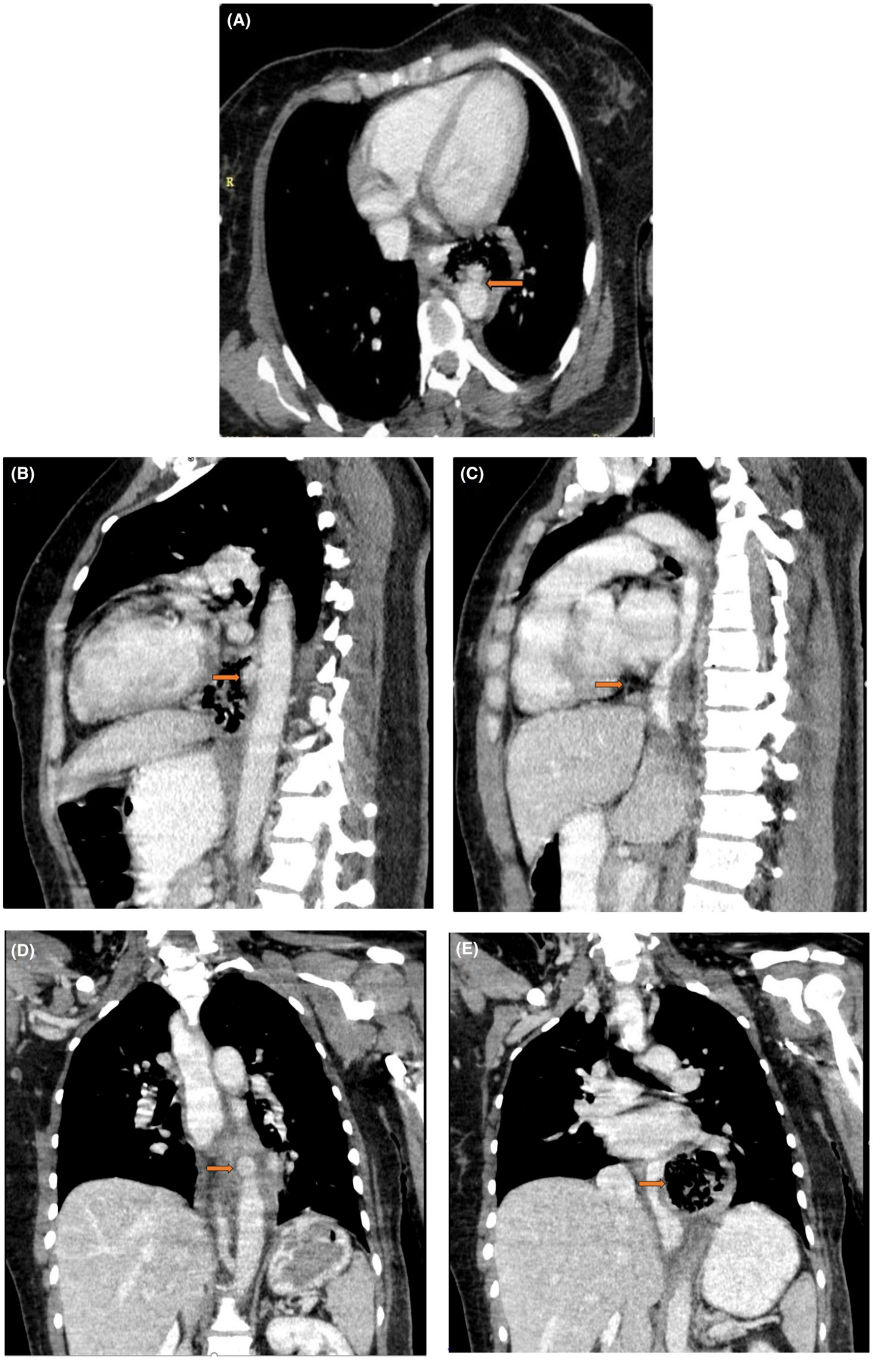
(A, B) Axial and sagittal reconstruction of the CT scan with IV and oral contrast. The images reveal an aortic wall outpouching with gas densities and fat stranding around it (arrow). (C) Sagittal reconstruction of the CT scan, indicating fat stranding and a deviation in the mid‐esophagus (arrow). (D, E) Coronal reconstruction of the CT scan, highlighting an aortic wall outpouching with gas densities and fat stranding around it adjacent to the esophagus (arrow). No obvious contrast extravasation was observed.

However, the CT scan revealed an outpouching with a diameter of 10 mm, with gas densities and fat stranding around it at the T7‐T8 vertebra level, indicating the presence of an aortic pseudoaneurysm, which likely suggested an aortoesophageal fistula and esophageal microperforations in that area. There was no evidence of pneumomediastinum, pneumothorax, or pneumoperitoneum. Additionally, there was no evidence of an obvious perforation in the esophagus (Figure 2).

Despite the absence of evidence of GI bleeding and coffee ground contents, and the patient's normal coagulation profile, the patient underwent supportive treatment and necessary preparations were made for surgical procedures. However, a few hours after the CT scan and before surgery, the patient experienced massive upper gastrointestinal bleeding. Despite supportive fluid therapy and blood transfusion, the patient progressed to hypovolemic shock. Unfortunately, despite the necessary resuscitation measures and adequate hemostasis, the patient ultimately experienced circulatory collapse and cardiac arrest, resulting in the patient's death.

## 3 DISCUSSION

Aortoesophageal fistula (AEF) is an abnormal connection between the aorta and esophagus. There are multiple causes of this condition, with the most common being thoracic aortic aneurysm, foreign body ingestion, and esophageal malignancies. Other common causes include complications after surgery, GERD, and tuberculosis.^5^ In a case report, Suzuki et al.^6^ reported the death of a Parkinsonian patient due to AEF caused by drug‐induced esophagitis. Ju et al.^5^ also reported a case of AEF in a patient with dermatomyositis, which was likely caused by drug‐induced esophagitis and NG tube irritation. Chao et al.^7^ reported the incidence of AEF as a result of inflammation caused by botulinum injection in the esophagus. In most cases, the pathological process involves the inflammatory destruction of the aortic wall with pressure necrosis of the soft tissue between the aorta and the esophagus, leading to the formation of a fistula between the aorta and esophagus.^8^

We suspect that the incident of AEF in our patient was the result of inflammation and esophageal wounds caused by continuous vomiting and endoscopic procedures. The esophageal wounds eventually deepened and passed through the wall of the esophagus, penetrating into the thoracic aorta.

Symptoms of AEF typically include chest pain and sentinel bleeding, followed by an asymptomatic period of several hours to several days (Chiari triad), after which the patient experiences massive bleeding.^9^, ^10^ However, not all patients exhibit these symptoms, with sentinel bleeding reported in only 50%–90% of cases.^2^ Our patient did not exhibit the typical symptoms of AEF (Chiari triad); the patient's only symptom was chest pain following the bougienage procedure. Due to the absence of the typical triad and a lack of clinical suspicion of AEF, diagnosis was delayed. Therefore, the key to diagnosing this event is to consider it as a potential endoscopic complication, as the triad is not present in many patients.

In a patient with chest pain, a chest x‐ray and ECG are typically performed. In a patient with AEF, mediastinal widening may be observed.^11^ However, there were no significant changes in the chest x‐ray evaluation of our patient.

In the study by Saers et al.^12^ only 38% of AEF cases were diagnosed during endoscopy. Therefore, endoscopy is not a reliable modality for diagnosing AEF. As in our case, endoscopy was not useful in diagnosing this complication. However, classic endoscopic findings that suggest the diagnosis of AEF include pulsatile bleeding and a submucosal mass with an adherent clot. The esophageal mucosa may also appear blue‐gray due to a submucosal hematoma.^1^

On the other hand, due to the temporary blockage of the fistula with a clot, angiography is not a suitable modality for diagnosing AEF, as contrast extravasation may not be present.^8^, ^13^

In Saer et al.'s study, it was demonstrated that CT scan with IV contrast was useful in diagnosing AEF in more than 50% of patients. Therefore, CT scan with IV contrast is the most favorable modality for patients with suspected AEF.^12^ Typical findings on a CT scan include the presence of ectopic gas densities around the aortic lumen and loss of the periaortic fat plane. Extravasation of contrast material from the aorta to the esophagus is a characteristic but rare finding.

Mycotic aneurysm is one of the causes of AEF. The presence of ectopic gas, aortic bulge, and periaortic fat stranding in association with leukocytosis, even without the presence of fever and positive blood culture, can suggest it.^2^ In our patient, these findings were also present, along with leukocytosis of 14.6 × 10^3^ (PMN dominant) and a C‐reactive protein level of 46 mg/L, which can suggest the possibility of an underlying mycotic aneurysm. Mycotic aneurysm is caused by an infection of the aorta with microorganisms, and in a patient with esophageal perforation, mediastinal infection, and invasion of the aortic wall by infectious agents can lead to this condition.^14^ The CT scan performed on our patient also revealed evidence of microperforation of the esophagus and resulting gas densities.

Treatment methods for AEF include surgery and thoracic endovascular aortic repair (TEVAR).^15^ TEVAR has recently been recognized as a bridging therapy for patients with AEF who are in shock.^16^ In a patient who is in shock due to severe bleeding and requires emergency measures to control the bleeding, TEVAR is a good option to stabilize the patient's condition and reduce short‐term mortality. Once the patient's condition is suitable for AEF repair surgery (including aortic replacement, esophagectomy, and greater omentum wrapping), surgical procedures are performed.^17^, ^18^, ^19^

In summary, inflammation is the basis of fistula formation between the aorta and esophagus. Endoscopic procedures and resulting inflammation can also lead to this condition. It is noteworthy that in this case report, endoscopy appears to have caused AEF, which has not been previously reported in the literature. Typically, the formation of EAF is not secondary to endoscopic procedures in the case reports published so far. Therefore, considering this diagnosis as a potential complication of endoscopic procedures can aid in early diagnosis and management. The occurrence of AEF may present with nonspecific symptoms and without the classic Chiari triad. Additionally, in most cases, endoscopy does not reveal any specific findings in favor of AEF. Therefore, the best modality for diagnosing AEF is CT scan with IV contrast injection.

## AUTHOR CONTRIBUTIONS

**Shokrollah Hafezeftekhari:** Investigation; resources; visualization; writing – original draft. **Farzaneh Khoroushi:** Conceptualization; supervision; validation. **Hossein Bozorgi:** Project administration; resources; validation; writing – review and editing.

## FUNDING INFORMATION

The authors did not receive any funding for this study.

## CONFLICT OF INTEREST STATEMENT

The authors declared no conflicts of interest.

## ETHICAL STATEMENT

This study was approved by the institutional review board committee.

## CONSENT

Written informed consent was obtained from the patient to publish this report in accordance with the journal's patient consent policy.

## Data Availability

The data that support the findings of this study are available from the corresponding author upon reasonable request.
